# A Rare Case of Massive Hematometra with a Tubo-ovarian Abscess in a 16-year-old Female

**DOI:** 10.7759/cureus.4845

**Published:** 2019-06-06

**Authors:** Georgios Tsatsaris, Zacharias Fasoulakis, Ioannis Papapanagiotou, Marianna Theodora, Emmanuel N Kontomanolis

**Affiliations:** 1 Obstetrics and Gynecology, Democritus University of Thrace, Alexandroupolis, GRC; 2 Obstetrics and Gynecology, National and Kapodistrian University of Athens, Athens, GRC

**Keywords:** imperforate hymen, hematometra, tubo-ovarian abscess, hymenectomy

## Abstract

Imperforate hymen is a congenital defect of the lower genital tract and specifically the vagina. The examination of a neonatal can be quite helpful to avoid a multitude of complications in puberty like hematocolpos and tubo-ovarian abscess.

We present the case of a 16-year-old who presented to the emergency department with fever (37.9° C), which was progressive the last two days, swollen abdomen, and pain in the lower abdomen. She also had a one-year history of cyclic abdominal pain. The patient had primary amenorrhea, the secondary sexual characteristics were normal for her age (Tanner III), and there was no family history of primary amenorrhea. The physical and ultrasound examination revealed an imperforate and bulging vaginal membrane and a multilocular adnexal mass, respectively.

Every doctor should suspect this medical condition when there is a triad of symptoms like cyclic lower abdominal pain, primary amenorrhea, and swollen abdomen. Early diagnosis of an imperforate hymen can prevent serious complications for young patients.

## Introduction

Hematometra is a medical condition that involves the collection of blood in the uterus. The most common cases occur due to uterine, cervical, and vaginal malformations; whereas, medical cases that cause obstruction of the cervical canal are far rarer. Imperforate hymen is a rare malformation of the vagina and a possible reason of hematocolpos and hematometra in puberty. This genital tract malformation has an incidence of 0.05% to 0.1%. This sporadic condition usually presents itself with the presence of a mass, lower abdominal pain, delayed menarche, and bulging vaginal membrane. Some of the complications of this medical condition include pelvic infection with tubo-ovarian abscess, obstructive acute renal failure, peritonitis, endometriosis, hematosalpinx, and constipation. The treatment of these patients is surgical, and the standard procedure is hymenectomy. It is of great significance that the surgeon should conserve the virginity of the young girl, so the incision should be made centrally [1]. The best time for this surgical intervention is still undetermined. This medical condition must be differentiated from vaginal septum, vaginal agenesis, vaginal and hymenal cyst. This study presents the case of a teenage girl, who came to the emergency department with acute abdominal pain, swollen lower abdomen, fever, and a history of primary amenorrhea.

## Case presentation

**Figure 1:**
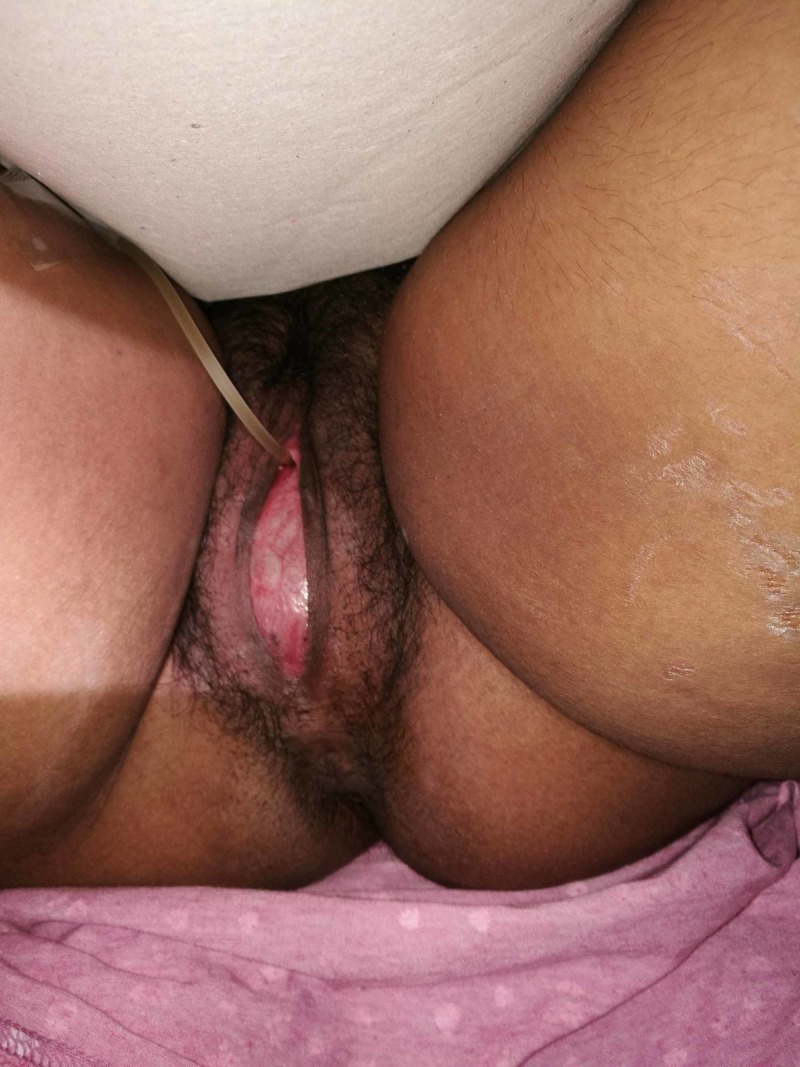
Imperforate and bulging membrane

Herein, we study the case of a 16-year-old, who came to the emergency department with fever (37.9 °C), progressive over the last two days, swollen abdomen, and pain in the lower abdomen. She also had a history of cyclic abdominal pain for almost one year. The patient had primary amenorrhea, the secondary sexual characteristics were normal for her age (Tanner III), and there was no family history of primary amenorrhea. During the physical examination, a large mass, dull to percussion, was found. The gynecological examination revealed an imperforate and bulging vaginal membrane (Figure 1).

**Figure 2:**
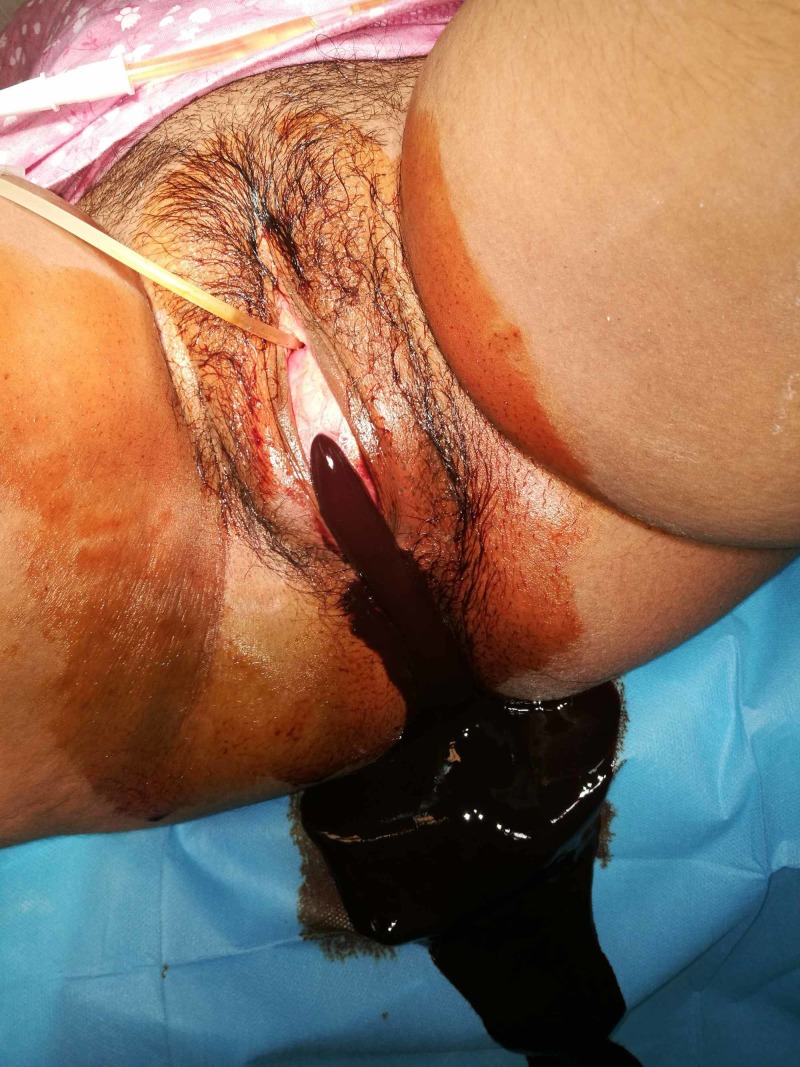
Hymenectomy

It was possibly a diagnosis of hematometra. The transabdominal ultrasound showed a 12-week gravid in size uterus, full of 1L low level echoed fluid. The ureters and kidneys were normal. The patient΄s lab tests showed a hematocrit level of 33%, a white blood cell (WBC) count of 41.333/mm3, platelets (PLTs) were 300,000/mm3 and also a high C-reactive protein (CRP). The values of urea, creatinine, and electrolytes had no deflection from the normal ones. Τhe ultrasound showed a multilocular adnexal mass with debris, septations, and irregular thick walls of 6 x 8.2 x 9.6 cm in size, located in the left adnexal region [2]. There was no collection of fluid in the Douglas pouch or any other suspicious findings. Additionally, a magnetic resonance imaging (MRI) was ordered [3]. The hematometrocolpos was clearly seen. The mass on the left ovary was thick-walled, filled with fluid and hypointense signal in T1 and typically heterogeneous in T2 series. The differential diagnosis were pelvic malignancy, pelvic endometriosis, pelvic hematoma, ovarian torsion, inflammatory bowel disease, and ectopic pregnancy [4]. But the diagnosis of tubo-ovarian abscess was more obvious because of the girl΄s symptoms, that is, fever and abnormal values of WBC and CRP. The right adnexal region seemed completely normal, without any suspicion of infection. The evacuation of the blood from the uterus with a central incision of the hymen under general anesthesia was the first priority. A large amount of brown-coloured blood was drained from the vagina and the uterine cavity (Figure 2).

Four absorbable sutures (Vircryl 2-0) were applied on the edges of the membrane. The Foley catheter, which is placed before the operation, was then removed. For the first 24 hours, 1.2 grams of amoxicillin/clavulanic acid was administered twice a day. At the same time, clindamycin 900 mg was administered by intravenous (IV) infusion every eight hours and gentamicin 2 mg/kg bolus, and then 1.5 mg/kg every eight hours were given. The CRP did not decrease after 72 hours properly, and we decided to perform an urgent laparotomy. The mass in the left adnexa was removed on the third postoperative day after the hymenectomy, without any complications. The operation lasted one hour and the blood loss was insignificant. During laparotomy, a mass on the left ovary was observed, which contained 20 ml of pus. Fortunately, it had not ruptured and was removed successfully. The patient recovered after the operation and continued her treatment with antibiotics intravenously for two days. Then, the patient was discharged on the fifth post-operative day (second post-operative day post laparotomy). She continued doxycycline per os at 100 mg twice a day for the next two weeks.

## Discussion

As we have already mentioned, imperforate hymen is a condition in which the symptoms usually appear in puberty. It appears mostly as primary amenorrhea, cyclic abdominal pain, hematocolpos, and hematometra [5-6]. Some of the main complications of untreated imperforate hymen include tubo-ovarian abscess, renal failure, hematosalpinx, endometritis, peritonitis, endometriosis, constipation, and urinary tract infection [1]. Some potential adverse consequences and complications are the risk of mortality, if left untreated, loss of an ovary, and diminished fertility. Most cases of tubo-ovarian abscesses are usually found in sexually active girls, especially in puberty. So, it seems that the collection of blood in the uterus is a source of microorganisms and a possible reason of pelvic inflammatory disease [7]. The above symptoms should concern the clinician so that he can take great measures to avoid complications. In a newborn, the pelvic examination can reveal an imperforate hymen or other malformations, due to the absence of mucus at the posterior commissure of the labia majora [8]. Due to the estrogen stimulation from the mother to the female fetus, the mucus accumulates in the vagina causing a bulging membrane that can easily be detected by the clinician. But, usually, the diagnosis of cases like this is delayed and is discovered in puberty [9]. Preservation of virginity should be taken into serious account, as virginity is cherished by many religions.

## Conclusions

Imperforate hymen is a birth defect of the lower genital tract. Every doctor should suspect this medical condition when there is a triad of symptoms like cyclic lower abdominal pain, primary amenorrhea, and swollen abdomen. The earlier the doctor detects this malformation, the fewer the complications will be for a young patient. Complicated cases like this are certainly unusual, and the early recognition of an abscess of the adnexa clinically and ultrasonographicaly is important, in order to prevent the associated catastrophic effects on the patient΄s health.

## References

[1] Bakos O, Berrghind L (1999). Imperforate hymen and ruptured haemosalphinx: a case report with a review of literature. J Adolesc Health.

[2] Jeong W, Kim Y, Song S (2019). Tubo-ovarian abscess: CT and pathological correlation. Clin Imaging.

[3] Kinay T, Unlubilgin E, Cirik D, Kayikcioglu F, Akgul M, Dolen I (2019). The value of ultrasonographic tubo-ovarian abscess morphology in predicting whether patients will require surgical treatment. Int J Gynaecol Obstet.

[4] Sieberg R, Tenhumen A, Yslostatlo OP (1985). Diagnosis of mucocolpos and hematocolpos by ultrasound: two case reports. J Clin Ultrasound.

[5] Adhikari S, Blaivas M, Lyon M (2019). Role of early bedside transvaginal ultrasonography in the diagnosis of tubo-ovarian abscess in the emergency department. J Emerg Med.

[6] Rana A, Gurung G, Begum S, Adhikari S, Neupane B (2019). Hysterectomy for hematometra in a 15-year-old mentally handicapped girl with congenital cervicovaginal agenesis and concomitant ovarian adenoma. J Obstet Gynaecol Res.

[7] Weström L (2019). Incidence, prevalence, and trends of acute pelvic inflammatory disease and its consequences in industrialized countries. Am J Obstet Gynecol.

[8] Temizkan O, Kabil Kucur S, Agar S, Gozukara I, Akyol A, Davas I (2019). Virginity sparing surgery for imperforate hymen: report of two cases and review of literature. J Turk Ger Gynecol Assoc.

[9] Al Ghafri A, Fida A, Al-Gharras A (2019). Obstructed hemivagina and ipsilateral renal anomaly (OHVIRA) syndrome. Oman Med J.

